# Sarcopenia: imaging assessment and clinical application

**DOI:** 10.1007/s00261-021-03294-3

**Published:** 2021-10-23

**Authors:** Vito Chianca, Domenico Albano, Carmelo Messina, Salvatore Gitto, Gaetano Ruffo, Salvatore Guarino, Filippo Del Grande, Luca Maria Sconfienza

**Affiliations:** 1 Clinica di Radiologia EOC IIMSI, Lugano, Switzerland; 2 Ospedale Evangelico Betania, Napoli, Italy; 3 IRCCS Istituto Ortopedico Galeazzi, Milano, Italy; 4 Sezione di Scienze Radiologiche, Dipartimento di Biomedicina, Neuroscienze e Diagnostica Avanzata, Università degli Studi di Palermo, Palermo, Italy; 5 Dipartimento di Scienze Biomediche per la Salute, Università degli Studi di Milano, Milano, Italy; 6 IRCCS Multimedica, Milano, Italy

**Keywords:** Body composition, Imaging, Sarcopenia, Muscle

## Abstract

Sarcopenia is a progressive, generalized skeletal muscle disorder characterized by reduction of muscle mass and strength. It is associated with increased adverse outcomes including falls, fractures, physical disability, and mortality, particularly, in elderly patients. Nowadays, sarcopenia has become a specific imaging biomarker able to predict clinical outcomes of patients. Muscle fibre reduction has shown to be an unfavourable pre-operative predictive factor in patients with cancer, and is associated with worse clinical outcomes in terms of postoperative complications, morbidity, mortality, and lower tolerance of chemoradiation therapy. Several imaging modalities, including dual-energy X-ray absorptiometry, CT, MRI, and US can be used to estimate muscle mass and quality to reach the diagnosis of sarcopenia. This article reviews the clinical implications of sarcopenia, how this condition can be assessed through different imaging modalities, and future perspectives of imaging of sarcopenia.

## Introduction

Sarcopenia has become the focus of research programmes worldwide because of its rising prevalence due to population ageing [1]. In particular, according to a systematic review, including different populations, the prevalence is higher in subjects living long term in residential care (14–33%) compared with elderly people living in the community (1–29%) [1]. Furthermore, worldwide prevalence of sarcopenia is expected to increase from 50 million people in 2010 to around 200 million in 2050 [2]. For this reason, sarcopenia is considered a relevant clinical and economic issue for public healthcare and was estimated to cost around $18.5 billion dollars in the 2000s in the United States both for direct (hospitalization) and indirect costs (nursing home admissions and home healthcare expenditure) [3].

## Definition and pathophysiology

Sarcopenia, described for the first time in the 1980s, is defined at present according to the European Working Group on Sarcopenia in Older People (EWGSOP), as a “syndrome characterized by progressive and generalized loss and changes of skeletal muscle mass and strength” that occur not only in elderly patients but also in younger subjects secondary to other pathologic conditions [4, 5]. Although the loss of muscle mass and muscle strength are distinct but interdependent factors, the loss of strength occurs faster than loss of muscle mass and seems to be a more important risk factor for adverse outcomes [6]. A multifactorial pathophysiological mechanism is the basis of sarcopenia, including genetic factors, decreased physical activity, poor nutrition, hormonal dysregulation, decreased D vitamin levels, inflammation, and cachexia due to chronic illness or cancer [7]. Ageing determines the imbalance between muscle protein anabolic and catabolic processes [8]. The loss of skeletal muscle mass and cellular changes are due to a reduction in size and number of myofibres [9, 10] and these events are partly due to the transition of muscle fibres from type II to type I with age, together with muscle fat infiltration (“myosteatosis”) [11]. Notably, type I and type 2 muscle fibres differ in many aspects (fibre size, force generation, resistance, contraction speed, etc.) with the former supporting long and resistance activities, while the latter being essential for fast and powerful motor activities. Changes in neurological signalling and control mechanisms also have a crucial role in the progressive loss of muscle function due to the increase of muscle fibre denervation resulting in downregulation of the anabolic pathway [12]. Recent studies demonstrate that some changes involve the peripheral nervous system such as motor unit loss, axonal atrophy, and demyelination caused by oxidative damage to proteins and lipids, and modified transmission of impulse through the neuromuscular junction [12, 13]. In addition, recent studies focussed on the strict interaction occurring between muscle, bone, and fat tissues and pointed out a new pathologic entity called “osteosarcopenia” or “osteosarcopenic obesity” defining combined phenotypes [14–16]. However, a consensus regarding the definition of “sarcopenic obesity” has not yet been established, and how muscle strength should be defined to make a diagnosis in these patients remains unclear [4, 17, 18].

## Clinical impact of sarcopenia

The concept of “muscle as a secretory organ” has revolutionized the idea of muscle function. Although skeletal muscle was previously considered a structure with a mere mechanical function, it has been demonstrated that muscles have various functions, both metabolic, endocrine, and neurological [19]. As an example, the relationship between sarcopenia and osteoporosis can be explained by the hyper-expression of myostatin that is a myokine with a crucial role in osteoclast formation, bone mass, and muscle mass [20, 21]. Other important hormones that take action in this relationship include growth hormone, insulin-like growth factor-1, sex hormones, and vitamin D, which decrease with increasing age thus contributing to the development of “osteosarcopenia” [22]. Of note, sarcopenia seems to increase the risk of falls, thus increasing the risk of fractures in patients already at higher risk due to their osteoporotic condition [16].

**Fig. 1:**
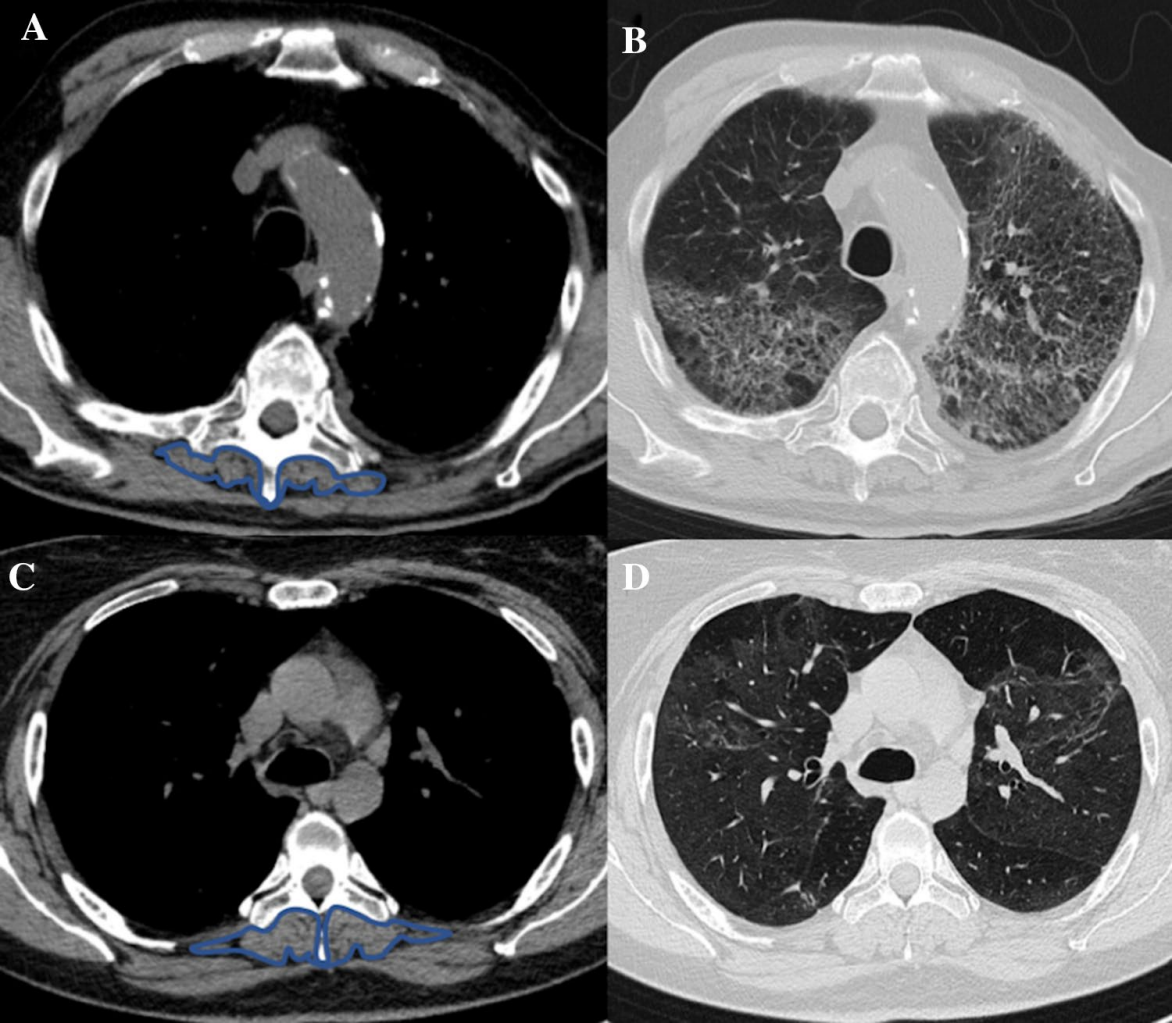
Computed tomography images of two patients affected by COVID-19 pneumonia. Images **A** and **B** refer to a sarcopenic male patient (skeletal muscle index of 42 cm^2^/m^2^) with severe parenchymal disease extent, while images **C** and **D** refer to a non-sarcopenic female patient (skeletal muscle index of 55 cm^2^/m^2^) with minimal parenchymal involvement

In the respiratory system, sarcopenia leads to an increase in morbidity and mortality in chronic diseases such as cancer and chronic obstructive pulmonary disease [23]. Recently, sarcopenia has been also reported as an independent factor associated with intensive care unit admission and mortality in patients hospitalized for COVID-19 pneumonia (Fig. 1) [24]. In inflammatory bowel disease, sarcopenia has a prevalence ranging from 27 to 61% [25], and is proven to be a negative prognostic factor for intestinal resection, especially in patients with Crohn’s disease [26]. In the setting of rheumatic disorders the development of sarcopenia may reach 20% due to the underlying proinflammatory state, pain, and subsequent reduced muscle use [27].

In the endocrine system, sarcopenia is a potential cause and consequence of the diabetes mellitus [22]; the possible explanation is that patients present significantly lower skeletal muscle mass resulting in reduced capacity for glucose disposal [28]. In patients with chronic pancreatitis, malnutrition is a common sequela that alters body composition resulting in sarcopenia [29].

In several gastrointestinal and pelvic malignancies, including oesophagus, stomach, liver, pancreas, bladder, and more, sarcopenia is known to adversely affect outcomes [30, 31]. Further, patients with reduced muscle mass have altered pharmacokinetics which cause a relative overdose in chemotherapy with consequently higher prevalence of side effects and therapy interruptions [32]. In addition, sarcopenia shows direct correlation with higher risk of post-surgical complications [33–36].

## Non-imaging diagnostic techniques

Physical performance can be evaluated with a series of physical exercises taking 10–15 min to evaluate the function of lower limbs using a combination of gait speed test, repeated chair rise time, and balance tests [37]. If physical test values are below the reference values proposed by the society definitions, sarcopenia should be suspected. *Anthropometry* is a cheap and non-invasive technique for assessing the size, proportions, and composition of the human body; it is the most common approach for recording body composition and includes body mass index, skinfold thickness, and body circumference (waist, thigh, and calf) [38]. *Bioelectrical impedance* analysis is a painless and non-invasive technique that involves the application of a low-intensity electrical current through the body. The parameters obtained include resistance, which measures the impedance after the passage of current through intracellular and extracellular fluids, and the reactance, which reflects the impedance of cell membranes [39]. Virtually, all electrical impulses in the region of interest travel through the muscle, with changes in resistance and reactance reflecting variations in the muscle tissue itself [40]. It should be taken into account that measurement errors may be due to changes in hydration, soft-tissue oedema, exercise status, or food intake [41]. However, the equipment for bioelectrical impedance analysis is not widely available and is not presently suitable for clinical application. Further, biochemical and laboratory markers such as the urine measurement of deuterated creatine (D3-creatine) have shown concordance with MRI but the clinical use is still limited [22].

## Imaging diagnostic techniques

### Dual X-ray absorptiometry (DXA)

**Fig. 2:**
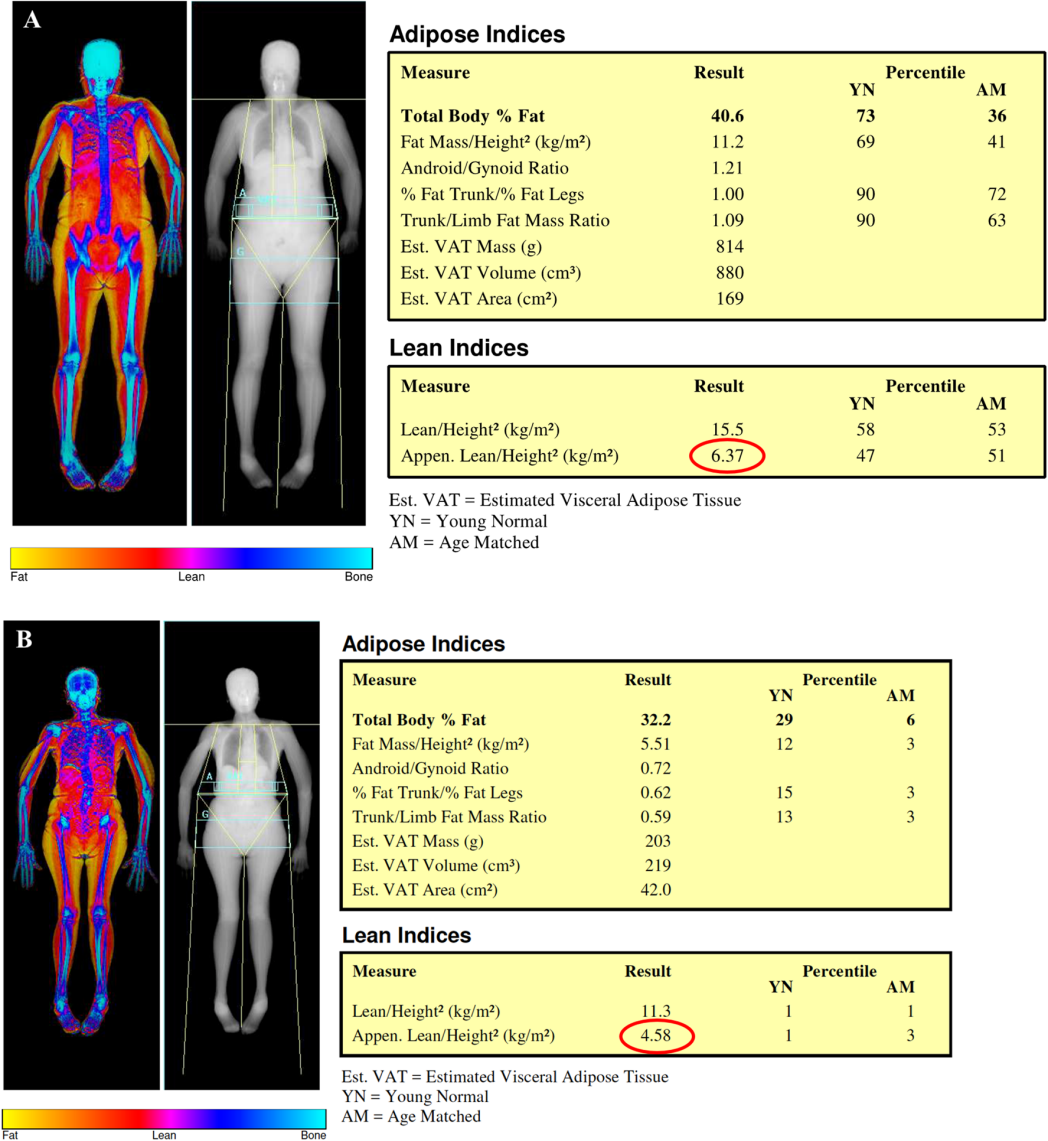
Dual-energy X-ray absorptiometry body composition scan of two female patients. Image **A** shows a 40-year-old woman without sarcopenia, with a value of Append. Lean/Height^2^ (ALMI index, see the red circle) of 6.37 kg/m^2^ (normal values > 6.0 kg/m2 for women according to EWGSOP2 [5]). Conversely, image **B** shows a 84-year-old woman with a DXA-based diagnosis of sarcopenia and a very low value of Append. Lean/Height^2^ (see red circle, ALMI index = 4.58 kg/m^2^)

DXA is the most commonly used imaging technique for body composition estimation especially in non-hospitalized patients [42]. It consists of a whole-body scan performed with an emitting X-ray source at two different energy levels (40 and 70 keV) (Fig. 2). The radiation dose varies depending on models and manufacturers, but it is usually around 5 µSv for whole-body acquisition, which makes DXA a safe option for body composition follow-up [40]. DXA allows the measurement of lean mass (LM), fat mass (FM), and bone mineral content (BMC) simultaneously [43]. LM measurement is an estimation of all non-fat/non-bone tissues. Notably, a strong correlation has been reported in literature with LM and FM values measured on CT and MRI [44]. The sum of upper and lower limbs’ LM, defined as appendicular lean mass (ALM), is used to quantify muscle mass with DXA. This value is indexed to height, and appendicular lean mass index (ALMI = ALM/height^2^) is obtained. The recent EWGSOP guidelines suggest an ALMI < 6 kg/m^2^ in women and ALMI < 7.0 kg/m^2^ in men as diagnostic cut-off values to define low muscle mass [5].

One of the main advantages of DXA is the capability to provide concurrently information about body composition and bone status that can be essential when evaluating disorders like osteoporosis, obesity, and cachexia [45]. Nevertheless, DXA has some limitations that should be pointed out. It is not able to quantify intramuscular adipose (in cases of myosteatosis) which impedes muscle quality estimation. Furthermore, body thickness, hydration status, and pathologic status with water retention such as heart, kidney, or liver failure can affect DXA results [46]. Indeed, DXA may overestimate muscle mass in patients with extracellular fluid accumulation, due to its inability to differentiate between water and LM tissue [47]. Conversely, DXA may underestimate trunk and thigh fat mass, and may overestimate thigh muscle mass in obese patients [48]. Finally, radiologists and radiographers should be instructed regarding the correct exam procedure, including patient positioning, demographic data collection, and image analysis in order to avoid pitfalls and misinterpretation [49].

### CT

**Fig. 3:**
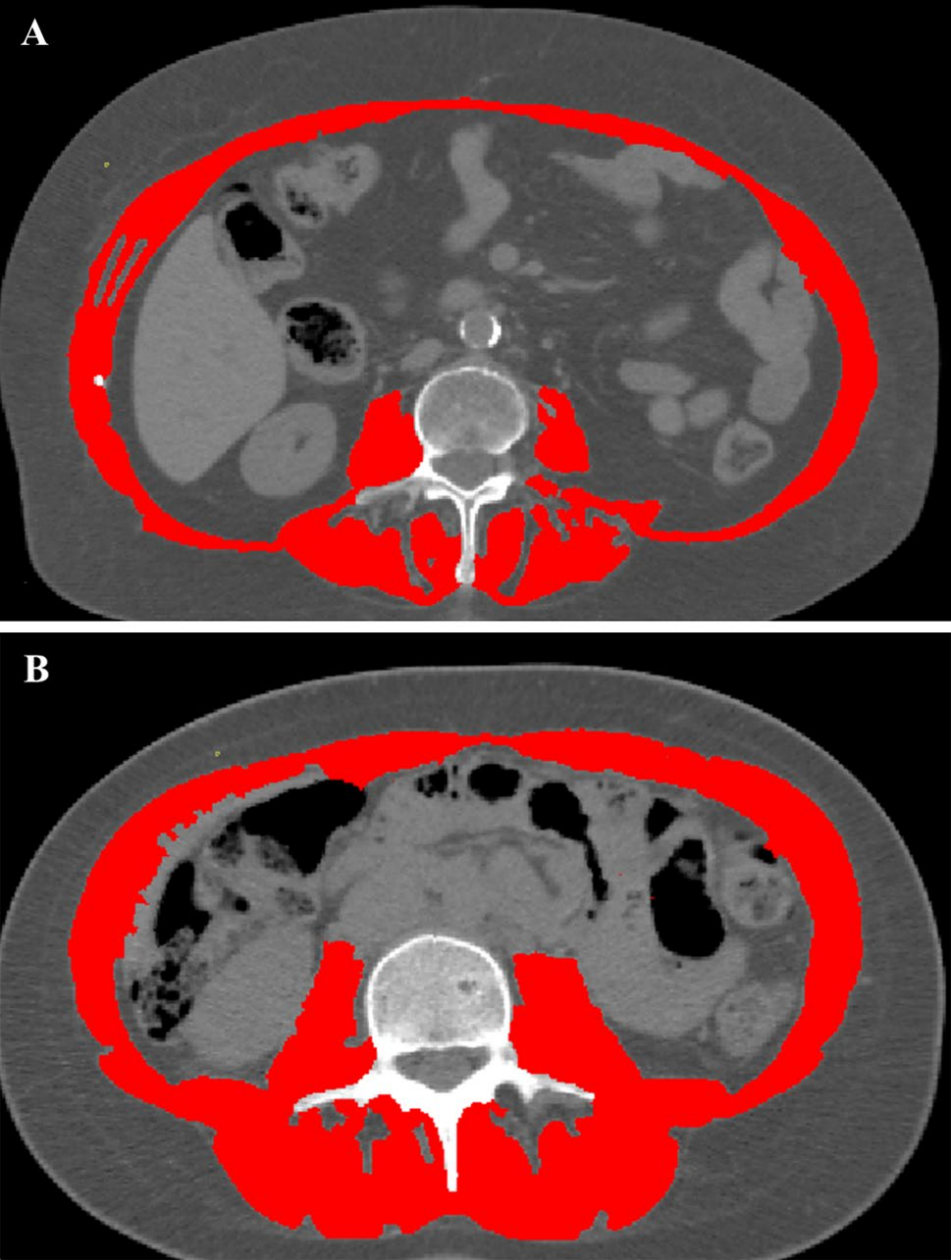
Computed tomography images of typical sarcopenic (image **A**) and non-sarcopenic (image **B**) patient. Skeletal muscle area (which is shown in red) is reduced in patient A, and this is especially visible in the paraspinal muscles which are larger and less infiltrated by intramuscular fat. A skeletal muscle index of 38 cm^2^/m^2^ and of 58 cm^2^/m^2^ were calculated using the CSA and height (CSA/height^2^) of patients A and B, respectively

**Fig. 4:**
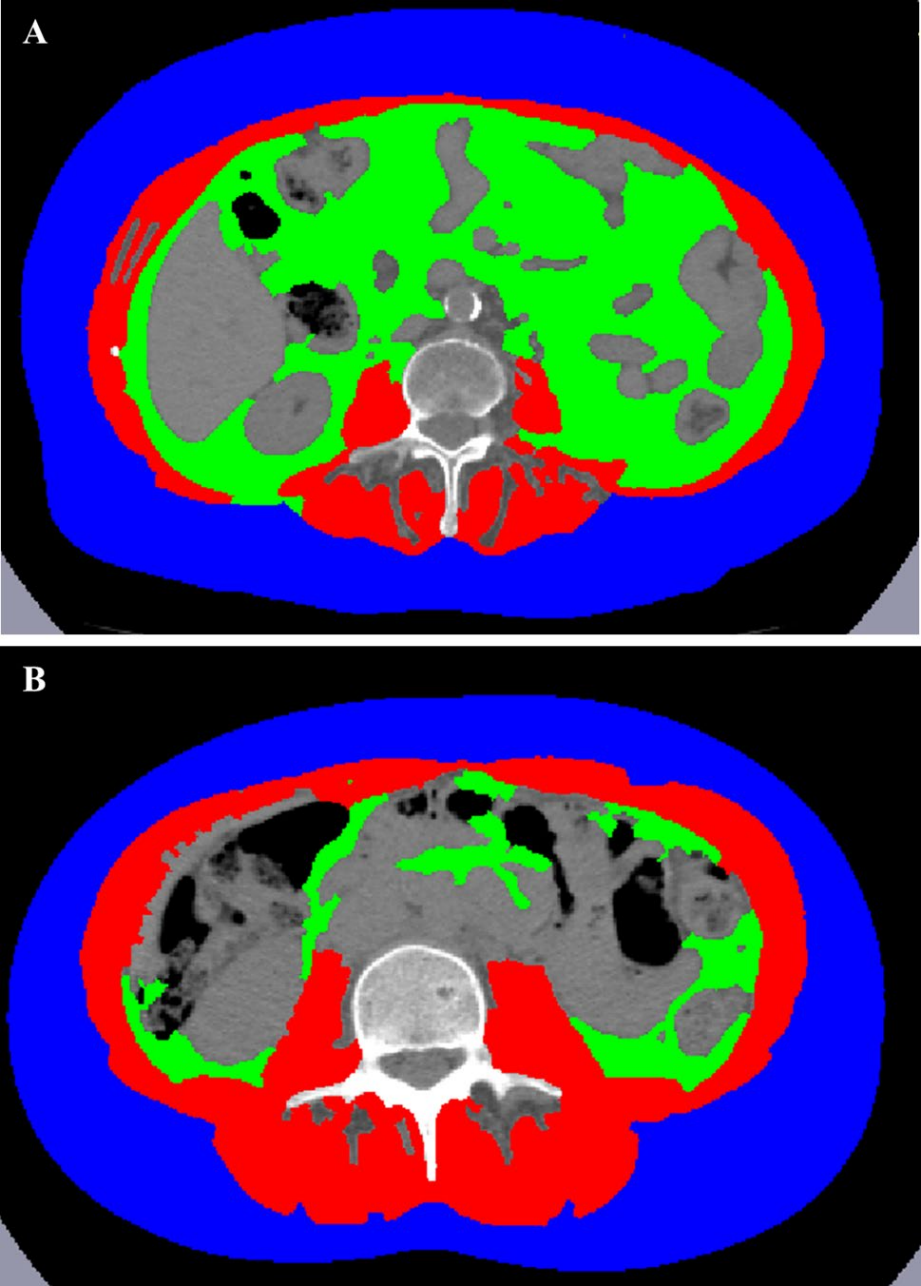
Body composition on computed tomography images of the same sarcopenic (image **A**) and non-sarcopenic (image **B**) patients of Fig. 2. Skeletal muscle area is shown in red colour, visceral adipose tissue area in green and subcutaneous adipose tissue in blue

CT is more and more used in research trials as routine diagnostic tool for the assessment of muscle quantity and quality, given that muscle density reduction is related to the degree of fat infiltration [7]. This imaging technique has the advantage of being performed for staging and follow-up of tumours and various other disorders, thus the assessment of sarcopenia is possible both in prospective and retrospective analysis without the need for additional scans. To assess muscle mass and quality, it is possible to draw a region of interest (ROI) traced to evaluate the cross-sectional area (CSA) and attenutation values of the skeletal muscles [50, 51]. A valid and accurate approach to estimate muscle and whole-body composition is to draw a ROI on a single axial CT slice to obtain the CSA applying standardized thresholds (− 29 Hounsfield HU/ + 150 HU) in order to include only muscle tissue during post-processing segmentation [52]; on the other hand, the thresholds of adipose tissue (− 190/ − 30 HU) are applied to assess the amount of intermuscular fat tissue. In most studies, the segmentation is performed at L3 or L4 level and ROIs include psoas muscles or all present muscles (paraspinous, psoas, abdominal) on the same slice (Fig. 3) [53]. On the same CT slice it is also possible to calculate information regarding the visceral adipose tissue and subcutaneous tissues obtaining a CT body composition (Fig. 4). It is important to highlight that non-contrast images should be used because muscle attenuation values increase after intravenous contrast media injection [54]. CSA is generally indexed for height (CSA/height^2^) to obtain the skeletal muscle index. A recent meta-analysis conducted by Amini et al. showed that the most common skeletal muscle index cut-off values reported in the literature for muscle mass assessment on CT at L3 range from 52 to 55 cm^2^/m^2^ for men and from 39 to 41 cm^2^/m^2^ for women [53]. However, L3 measurements are significantly different from those of other vertebral levels [55] and these cut-offs are only applicable on abdominal CT scans, excluding imaging of the chest (for example, for lung cancer screening) and of the pelvis without abdomen (in case of gynaecological cancer or hip fracture). In this regard, the skeletal muscle cut-offs from T10 to L5 reported by Derstine et al. extend sarcopenia evaluations to chest and pelvis examination to enable wider clinical applications of this imaging technique [56]. For this reason, other authors have tested the possibility to estimate muscle status around the hip to be used to predict outcomes after pelvis surgery [57]. Skeletal muscle and body composition evaluation using CT is shown in Figs. 3 and 4, respectively.

### MRI

**Fig. 5:**
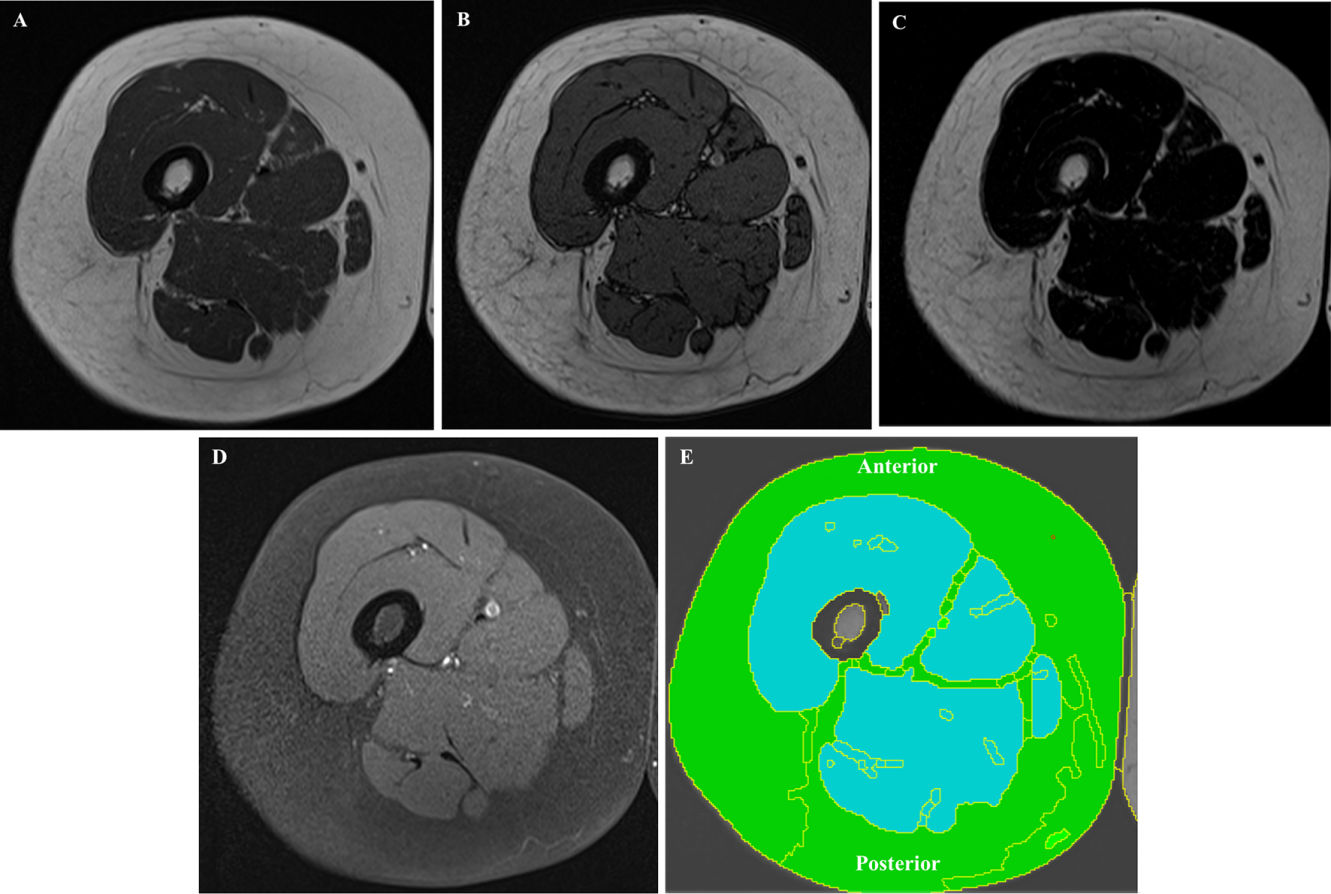
Dixon magnetic resonance sequence acquired on the right thigh of a 62-year-old woman. Axial T1W in-phase (image **A**), out-of-phase (image **B**), 100% fat images (image **C**), and 100% water images (image **D**). After post-processing, a semi-automatic segmentation can be obtained (image **E**) showing fat (green) and muscle tissue (turquoise) separately in the same slice

MRI enables to assess muscle composition by using several semi-quantitative or quantitative sequences without the need of ionizing radiations. Indeed, although MRI can be used to measure CSA of skeletal muscles, advanced MRI sequences allow to combine the estimation of body composition and the evaluation of muscle quality abnormalities, such as muscle disruption, oedema, fat infiltration (myosteatosis) or fibrosis (myofibrosis) [58], with the last two components increasing within muscles during ageing [59]. Different sequences can be used to assess sarcopenia:

- T2 mapping has been proposed as an imaging marker for fatty changes of skeletal muscle tissue [60] because it allows objective, comparable, and precise assessment of small changes in the muscle fibre composition [61]. Multi-echo sequences are used to generate T2 maps, by acquiring multiple echoes of the same image with pixel by pixel fitting of the T2 relaxation curve [62]. Measurements of T2 relaxation times can be used to identify pathological increase of T2 values of fat-infiltrated muscles in sarcopenic patients due to microstructural changes [63]. Currently, the T2 mapping in sarcopenia has been used only for research purposes and no cut-off values are available to differentiate sarcopenic from non-sarcopenic patients, thus its use in clinical practice still needs to be validated.
- MR spectroscopy is a functional non-invasive imaging technique that provides information regarding the biochemical processes within the body. There are theoretical advantages to multivoxel spectroscopy for sarcopenia evaluation because it can provide quantitative information related to a ROI due to the processing of several smaller voxels simultaneously, in comparison to single voxel spectroscopy, which represents the entire muscle belly, but additional evaluation is required [64]. However, multivoxel spectroscopy has rarely been used in sarcopenia and more clinical research will be required to clarify the actual role of this technique in sarcopenia [65].
- Dixon sequences overcome the limited applicability of MR spectroscopy due to inhomogeneity of fat muscle infiltration, thereby allowing precise quantitative assessment of sarcopenia [66] (Fig. 5). From a technical point of view, two- or multi-point Dixon sequences offer the possibility to obtain two separate “fat only” and “water only” images, through the acquisition of two or more echoes at different echo times [67]. The first acquisition is performed at an echo time when fat and water protons are in phase, while a second acquisition is then performed at an echo time in which the protons are out of phase [68]. Moreover, another advantage is the possibility to combine dixon with different types of sequences, both gradient and spin echo, and with different weightings (T1-w, T2-w or PD-w) [69]. Studies demonstrated that Dixon shows high accuracy in the detection and quantification of fatty replacement within muscles in dystrophic [70] or diabetes mellitus patients [71]. Despite these advantages, this sequence requires long acquisition time and is sensitive to metal artifacts in case of prostheses [68].
- Diffusion tensor imaging is an advanced MRI sequence, traditionally used for tracking track fibre course in the central nervous system that offers the possibility of detecting and quantifying muscle fibre fat replacement [72, 73]. Fractional anisotropy is a parameter, ranging between 0 and 1, used to quantify the directional orientation of water molecules as an indirect sign of microstructural fibre changes [74]. It seems that fat replacement presents a positive correlation with fractional anisotropy values in sarcopenic patients and negative correlation with apparent diffusion coefficient [75]. Further, diffusion tensor imaging tractography demonstrates the decrease in fibre length, number, and architecture, providing further quantitative information. This sequence is still under investigation in several studies as a promising non-invasive imaging tool to assess quantitatively muscle status and fat replacement, but its clinical value needs to be proven.

Although MRI is a promising technique in sarcopenia, with the advantages of no radiation exposure and excellent accuracy in measuring quantitative and qualitative parameters of skeletal muscle, it is still confined to the research stage with scarce application in clinical practice due to the high cost, lengthy acquisition, and lack of cut-off values and standardized protocols.

### US

**Fig. 6:**
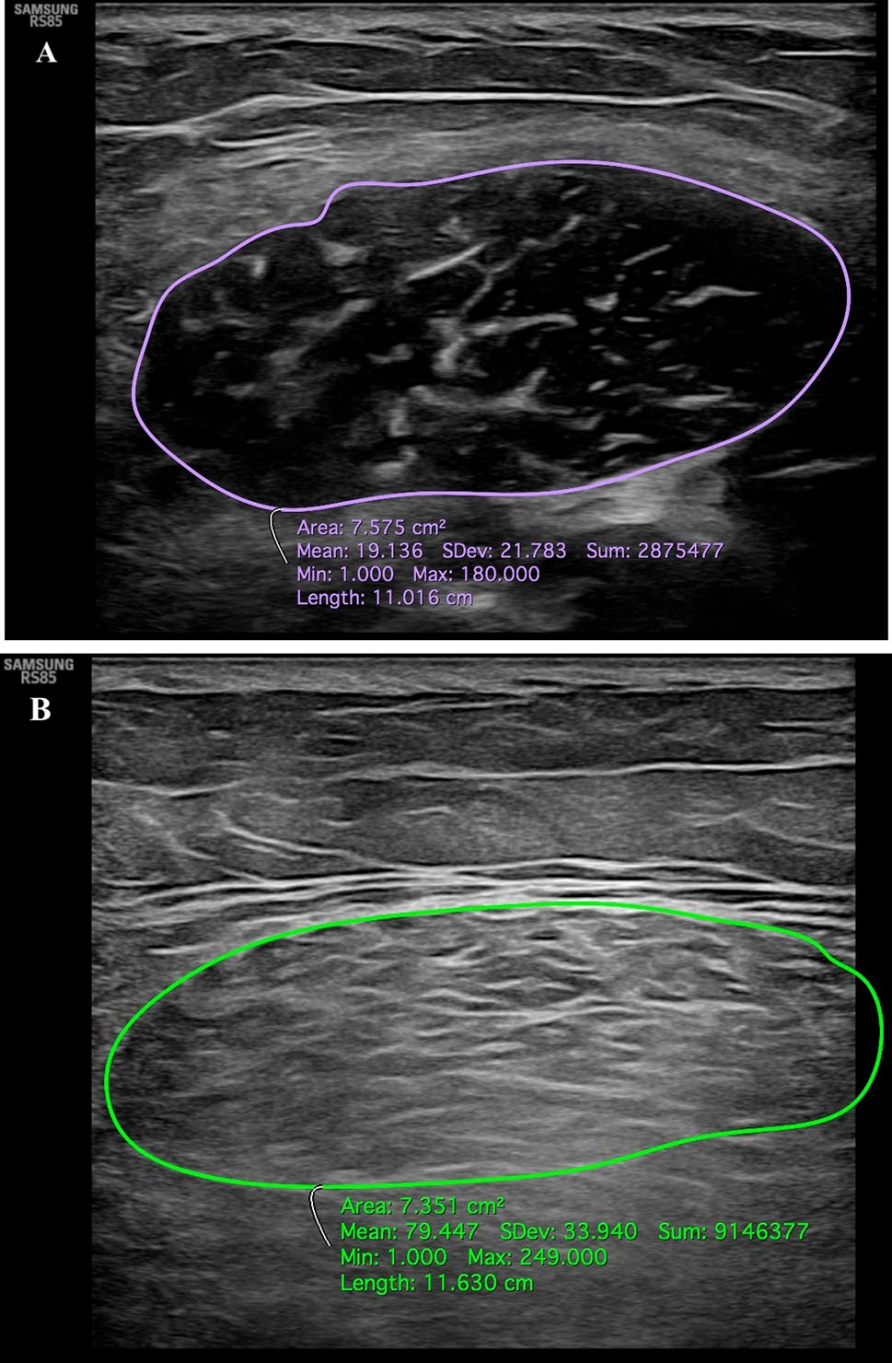
Ultrasound transverse images of the proximal third of the rectus femoris of two female patients with similar age and BMI. Image **A** shows the rectus femoris of 38-year-old runner (BMI = 24.2), while image **B** shows the rectus anterior of a 42-year-old patient (BMI = 25.1) who does not practice any physical activity. Despite a similar muscle trophism (CSA of patient in image **B** = 7.575 cm^2^, CSA of patient in image B = 7.351 cm^2^), an increased muscle belly echogenicity can be observed in the patient with limited physical activity due to fatty infiltration (image **B**)

US is a portable, inexpensive, non-invasive technique without ionizing radiations widely used to investigate musculoskeletal conditions [76]. It has already proven its worth in the assessment of muscle quality and quantity [77] as an accurate technique for the estimation of muscle properties showing strong positive correlation with DEXA [78], CT [79] and MRI-based measurements [80]. Previous studies showed good intra- and inter-rater reliability both in elderly people [81] and younger population [82]. US also provides information about muscle echotexture depending on the degree of intramuscular fat and connective tissue infiltration [83]. There are five components that can be easily measured when assessing muscle components in sarcopenic patients: muscle thickness, pennation angle, fascicle length, echo intensity, and CSA [84]. However, it is unclear which anatomical site is best for specific outcomes as a prediction of total skeletal muscle mass. The majority of studies have been performed on the quadriceps muscle, while few have focussed on others, such as postural muscles [83, 85, 86]. When measuring the maximal thickness and CSA of a muscle, it is advised to take the midpoint of the muscle between the tendons, and the point halfway between the medial and lateral border of the muscle belly [87] (Fig. 6). The pennation angle and muscle fascicle length must be assessed at rest and during contraction. These parameters are significantly associated with the loss of maximum force and shortening velocity of muscle fibres in sarcopenic patients [88]. The geometrical arrangement of muscles should be considered as it impacts on the generation of muscle force. The pennation angle can be calculated between the longitudinal axis of the muscle belly and its fibres. It is an important point given that the muscle force is related to its shortening velocity, so the slower shortening velocity of pennate muscle fascicles can lead to greater muscle force. Hence, the obliqueness of muscle fascicles and their rotation within the muscle belly enable them to be geared to slower speeds of contraction than the muscle belly, giving them higher force potentials. Structural changes associated with sarcopenia, including muscle size reduction and shortening of fascicles that become less pennate, have been associated with lower performance in elderly [85]. Notably, the anatomical CSA (ACSA), which is the CSA perpendicular to the longitudinal axis of the muscle belly, should be distinguished from the physiological CSA (PCSA), which is the CSA perpendicular to muscle fibres. The latter better describes the contraction properties of pennate muscles, indeed, while the ACSA and the PCSA are the same in non-pennate muscles, they differ in pennate muscles in which the ACSA underestimates the number of total fibres. In this setting, muscle strength is more closely correlated with PCSA, which is defined as the CSA of a muscle perpendicular to its fibres, because the former represents the maximum number of actomyosin crossbridges that can be activated during contraction [84, 89].

US elastography has been proposed as an investigative tool for muscle stiffness using shear-wave elastography and correlates with lower stiffness values in both lower and upper limb skeletal muscle in elderly [90]. Contrast-enhanced US has also been proposed as a US tool for assessing changes in microvasculature associated with sarcopenia but this point has been scarcely investigated in the previous studies [91]. Even though US shows promising results for the investigation of sarcopenia, the lack of standardized cut-off values strongly limits its validation for the clinical practice [92]. We have resumed in Table 1 the strengths and weak points of imaging modalities used to assess sarcopenia.

**Table 1:** Advantages and disadvantages of the main imaging tools for the assessment of body composition and mass/fat assessment

Technique	Strengths	Weak points	Measurements
DXA	• Fast acquisition • Widely available • Accurate and reproducible • Validated cut-off values • Minimal irradiation • Cheap	• Different results varying the densitometer brands • Dependent by hydration status • Bidimensional data	• Appendicular lean mass (ALM) •Appendicular lean mass index (ALMI)
CT	• Fast acquisition • Widely available • Accurate and reproducible • High spatial resolution	• Non-negligible irradiation • Cut-off values are still not used in clinical practice • Expensive • Time-consuming segmentation process	• CSA indexed by height^2^ (SMI) • Attenuation values
MRI	• Accurate and reproducible • No irradiation • High contrast resolution • Capability to identify muscle oedema and fat infiltration • Promising advanced sequences	• Long acquisition time • No validated cut-off values for sarcopenia • Expensive • Lower availability • Long post-processing of advanced sequences	• CSA indexed by height^2^ (SMI) • Fat content on DIXON • Experimental measurements of advanced sequences (ADC, FA, T2 relaxation time..)
US	• Fast acquisition • Widely available • No irradiation exposure • Cheap • Real-time imaging	• Scarcely reproducible • Poor accuracy • No validated cut-off values for sarcopenia	• CSA indexed by height^2^ (SMI) • Muscle thickness and echogenicity

## The role of artificial intelligence (AI) in sarcopenia

AI is a field of computer science dedicated to the creation of systems performing tasks that usually require human intelligence, thus allowing machines to better interpret huge amounts of data [93]. This is a group of different subfields but the most important areas in radiology are machine learning and deep learning [94]. With machine learning different algorithms are trained to recognize specific characteristics by learning patterns from datasets [95, 96].

In sarcopenia, AI determines an automated CT segmentation of muscle, bone, and adipose tissues, allowing a broad use of prospective or retrospective analysis of CT or MRI images [7, 97]. The data generated by software may be independent to the interobserver variability in sarcopenia quantification. Dong et al. analysed CT images of 99 patients with advanced non-small cell lung cancer, with 40 of them having been identified as sarcopenic using skeletal muscle CSA at the L3 vertebral level; the authors extracted 854 radiomic and clinical features from the skeletal muscle area at the 12th thoracic vertebra level and five optimal features were selected [98]. An automated muscle measurement on a single-slice chest CT at T12 vertebral level was used by Lenchik et al. to determine a relationship between muscle measurement with the rate of survival in a large patient population of 6.803 men and 4.558 women. Paraspinous muscles were segmented and the CSAs were recorded. The automated machine learning process found a significant relationship between lower paraspinous skeletal muscle area/density and worse patient survival [99].

Barnard et al. trained a convolutional neural network to perform muscle segmentation at T12 on a training dataset of 1875 single-slice CT images acquired on 4 different scanner manufacturers and tested their accuracy on 209 different CT images. The machine learning algorithm measurements of the CSA and the muscle attenuation of the left paraspinous muscle were compared with that of manual segmentation using Dice similarity coefficients and Pearson correlations. They found a favourable correlation of the machine learning algorithm compared to manual segmentation for measurement of paraspinous muscles with a Dice score of 0.94 [100]. AI could help segmenting skeletal muscles in a short time and as precisely as trained subjects. In the future, automated CT or MRI analysis will reduce the time-consuming segmentation process and will be used to classify patients at risk for sarcopenia to predict prognosis or to recommend follow-up and potential therapeutic interventions.

Recently, a different AI approach has been tested to assess body composition; Kim used radiomic features extracted from CT images to identify sarcopenia in non-small cell lung carcinoma patients reporting interesting results [101]. Indeed, the concept of radiomics, understood as the conversion of images to a huge amount of data, could be the future of imaging assessment of sarcopenia. To date, radiomics has been mostly used in oncology to predict the outcome of patients with cancers, but the potential use of radiomic features to facilitate the diagnosis of sarcopenia could be the subject of future investigations.

## Conclusion

Sarcopenia is a chronic disease with increasing prevalence among older adults. This condition is associated with worse outcome of several disorders with non-negligible economic burden of the healthcare system. Several imaging modalities can be used to assess sarcopenia, with the reduction of muscle mass on CSA being the main diagnostic finding. DXA is the most widely used imaging modality with defined cut-off values to identify patients affected by sarcopenia, while CT and MRI are mainly used for research purposes, despite they are reliable cross-sectional techniques with the ability to reveal both quantitative and qualitative changes in muscle mass. US is another imaging technique able to assess the muscle status, but still with a marginal role in sarcopenia imaging. The most recent studies on AI are mostly focussed on the reduction of the time-consuming process of muscles segmentation to measure CSA and muscle composition with the purpose to make easier to screen categories at risk of sarcopenia. Muscles are almost invariably included on diagnostic scans and radiologists must be aware of the prognostic role of sarcopenia as a more and more interesting imaging biomarker.
